# Thrive during a crisis: the role of digital technologies in fostering antifragility in small and medium-sized enterprises

**DOI:** 10.1007/s12652-022-03816-x

**Published:** 2022-03-22

**Authors:** Vincenzo Corvello, Saverino Verteramo, Isabella Nocella, Salvatore Ammirato

**Affiliations:** Department of Mechanical, Energy and Management Engineering, University of Calabria, Rende, Italy

**Keywords:** Antifragility, COVID-19, Digital technologies, Resilience, SMEs

## Abstract

The crisis triggered by the COVID-19 emergency is changing the competitive landscape by pushing companies to adapt to sudden change. Small and medium-sized enterprises (SMEs) that want to survive must innovate their business. Antifragility represents the capability of a system to absorb shocks and get better, allowing it to overcome a crisis and improve its performance. The use of digital technologies by enterprises is expected to play an important role in building antifragility. The aim of this paper is to study how digital technologies can contribute to the development of antifragility in SMEs. This study analyzed the responses to the COVID-19 pandemic crisis of six small and medium-sized enterprises located in Calabria, South of Italy. All the six enterprises have turned the crisis into a business opportunity developing new products, investing in marketing and communication, or starting new collaborations. The research identifies the factors leveraged by the investigated organizations that enabled this anti fragile behavior. They include slack financial resources, strategic agility, and relations with research institutions. The study highlights the positive impact of digital technologies in developing antifragility. Results were summarized into research propositions to be tested in future confirmatory studies. The findings of the study are useful for researchers interested in antifragility and digital technologies in SMEs. The results are also important for entrepreneurs and managers of SMEs, since they can support their decisions in terms of survival and transformation of their companies.

## Introduction

COVID-19 pandemic did not only represent a shock for small and large businesses: it has changed the competitive landscape and the business environment in general. The values and habits of the entire world population are changing, modifying products and services demanded as well as the way people work (Belghitar et al. 2021). Some firms will not survive the crisis, others will need to adapt to the new landscape. Digital technologies are expected to play a critical role in adapting to the new context and, consequently, for the survival of businesses, especially the small and medium-sized ones (Priyono et al. 2020).

SMEs are especially fragile in face of a crisis, due to the limited availability of financial, organizational, and human resources (Inekwen 2019; Herbane, 2019). In some cases, however, their leanness can represent an opportunity: in fact, SMEs can to convert their processes and capabilities, rapidly occupying new market niches (Ahn et al. 2018; Mahdad et al. 2020; Miroshnychenko et al. 2021). By networking with research institutions, startups, and other actors, they can innovate their business (Puliga et al. 2020).

Through their flexibility and rapid response capabilities, SMEs can provide the economy and society with solutions needed in the short term, to manage the emergency, and in the long term, to adapt to the new, post COVID-19 context. It is important, however, to understand what factors make the difference between organizations that succumb, and organization that recover or even thrive after a shock.

Resilience and antifragility are two concepts that have been frequently used to describe how firms survive and even thrive in unpredictable business environments. Resilience represents the capability to absorb shocks and, although temporarily changing, recover afterwards. Antifragility, instead, represents the capability of a system to absorb shocks and get better (Ramezani and Camarinha-Matos 2020).

While studies on resilience in organizations are numerous, research on antifragility is still limited. In particular, studies on antifragility in SMES are rare (de Bruijn et al. 2020). Antifragility is a highly desirable property, but how to develop it remains unclear (Chroust and Aumayr 2017).

Several factors are expected to contribute to antifragility. Some of these are internal to the company, others are external (Gimenez-Fernandez et al. 2020). Leuridan and Demil, for example, highlight how internal slack resources, in greater quantities than strictly necessary, facilitate the success of firms during a crisis (Leuridan and Demil 2021). Given the relative novelty of the phenomenon, however, what are the antecedents, both internal and external, of antifragility is still unclear. To fill this gap, this paper addresses the following research question:

RQ1: what internal and external factors have an impact on antifragility in SMEs?

As anticipated above, digital technologies are expected to play a critical role in the transformation and chances of success of SMEs after the COVIDCOVID-19 pandemics. The existing literature suggests that business companies are radically changing their way of working under the influence of digital technologies (Cappiello 2020). Existing evidence also suggests that adoption of digital technologies increases the resilience of firms during disruptive events (Autio et al. 2021). This leads to the idea that digital technologies play an important role also in building antifragility.

Studying how SMEs reacted to the emergency created by COVID-19 is important to understand how they develop and implement antifragility.

For what has been said above, this study also addresses the following research question:

RQ2: what is the role of digital technologies in creating antifragility in SMEs?

The paper is exploratory in nature. The topic under consideration is relatively new and requires qualitative investigations capable of capturing multiple factors that influence the phenomenon as well as the complex interrelations among these factors (Eisenhardt and Graebner 2007). To answer the two research questions, a multiple case study was carried out involving six Italian SMEs. These companies were selected because they transformed themselves during the pandemic to catch new business opportunities. One of the main outcomes of the study is the formulation of theoretical propositions related to the two research questions, to be tasted in future, confirmatory studies.

The paper is structured as follows: in Sect. 2 the literature on antifragility and its antecedents, with specific reference to digital technologies, is briefly summarized. In Sect. 3 the methodology for data collection and analysis is discussed, followed, in Sect. 4, by a description of the results. The final sections report discussion and conclusions, including implications, limits and directions for future research.

## Theoretical background

### Antecedents of antifragility in SMEs

Currently, organizations are increasingly exposed to unforeseen disastrous events (Petit et al. 2013), which are no longer an exception, but have become more and more frequent. The effects and consequences of these events are often unpredictable, causing major damage to many companies (Gotham and Campanella 2010). The capability to survive in a dynamic and turbulent environment is becoming very important as it is increasingly difficult to rely on traditional forecast-based approaches (Geldenhuys et al. 2020).

Antifragility is the ability to respond to a crisis by transforming the business model, possibly improving performance (Blečić and Cecchini 2019; Conz and Magnani 2020). Antifragility can manifest itself in the three main phases of crisis management (Ramezani and Camarinha-Matos 2020): *readiness*, that is the phase preceding the disaster; *response*, that is the actions implemented during and immediately after the crisis; *recovery*, or the actions that are implemented post-crisis. Whatever the specific manifestation of antifragility, past research suggests that there are several factors that contribute to its development: business intelligence (Pettit et al. 2013); flexibility, (Fiksel et al. 2015) lean structures Gotham and Campanella 2011).

Focusing on SMEs, crises can become an opportunity if these organizations have the ability to exploit the changes in the environment to increase their competitiveness (Mendoza et al. 2018). But to achieve this kind of success, they need to acquire specific resources and skills (Máñez et al. 2015; Mahdad et al. 2020). The characteristics of SMEs, such as flexibility and adaptability, are crucial to respond to a crisis because they make the decision-making process faster, thus obtaining a reduction in response times (Branicki et al. 2018). SMEs are generally used to working in conditions of uncertainty because, for example, of their limited financial and human resources. This entrepreneurial attitude makes them more comfortable in conditions of uncertainty compared to large organizations. This can be an advantage for SMEs, as they are more easily able to perceive the crisis as an opportunity (Branicki et al. 2018).

The factors that make a SME able to adapt to varied external conditions can be grouped in internal and external (Gimenez-Fernandez et al. 2020).

Internal factors mean resources and capabilities controlled by a SME which make it able to adapt to changing environmental conditions. For example, Leuridan and Demil (2021) underlined the role of internal slack resources in facilitating the success of firms during a crisis (Klein and Todesco (2021) have found that lack of financial resources contributed to make SMEs more fragile during the COVID-19 crisis. On the other hand, Lafuente et al. (2017) have found that an easier access to financial resources has a positive impact on resilience of SMEs, especially in the construction sector.

Besides resources, also skills and capabilities have been found to affect resilience and antifragility in past research. Flexibility, technical knowledge, creativity, are crucial when facing crisis and in general negative external events (Frare and Beuren 2021). Ramezani and Camirinha-Matos (2020) summarized the internal skills which facilitate the development of antifragility: creativity, understood as the ability to grasp opportunities during a crisis/disaster; adaptability or flexibility, meaning the ability to change in response to major changes or disruptions; transformability, that is the ability to transform process, structure, and behavior to survive during a crisis. All these skills can be associated to the concept of strategic agility, that is the ability to identify and adapt structures and processes to new opportunities (Soni et al. 2014; Zitzmann 2014; Carvalho et al. 2012; Wieland and Wallenburg 2012). Agility has been found to facilitate the success of SMEs in complex environments (Bianchi et al. 2017; Troise et al. 2022). As a consequence, it is expected to facilitate antifragility in SMEs facing large crises.

Graça and Camarinha-Matos (2017), however, suggest that the organizational level might be inadequate to understand antifragility. The most adequate level is the business ecosystem level. In their study on the textile industry, Pal and colleagues have found that resilience depends as much on the internal resources of an organization as on its network of external relations (Pal et al. 2014). With reference to supply chains, Asamoah and colleagues have found that external networking has a strong positive impact on the success in overcoming crises for SMEs (Asamoah et al. 2020).

In summary, the existing literature suggests that the ability of SMEs to face a crisis depends on a mix of resources and capabilities, both external and internal. Financial slack resources, internal skills and external network have been found to play a critical role. However, since existing studies are mainly focus on resilience, more research is needed on the specific antecedents of antifragility in SMEs.

### Digital technologies, resilience, and antifragility in SMEs

Digital technologies include categories such as the Internet of Things (IoT), Big Data analytics, Artificial Intelligence, Advanced Tracking and tracing technologies, Wearables and Additive Manufacturing (Buyukozka and Gooçer 2018). They increasingly impact on the development of small and large organizations (Oukil 2011) and influence the behavior of managers and entrepreneurs (Andriole 2017; Saritete et al. 2021).

The ever-increasing development and diffusion of digital technologies does not only affect digital companies (Ammirato et al. 2019), but all types of businesses (Kraus et al. 2019; Nambisan 2017).

Digital technologies have a huge impact on SMEs as well (Li et al. 2018). They facilitate the exchange of goods, services, or social currency, enabling value creation for all the actors through the digital landscape (Parker et al. 2016). The internal context of SMEs is being transformed (Lee 2016; Neirotti et al. 2019). Rosenblat and Stark (2016) have shown that digital technologies positively influence the employee-employer relationship in terms of autonomy and control, as they bring greater flexibility (Depaoli et al. 2020). Digital technologies have a direct impact on the work of entrepreneurs and managers, which in turn affects SMEs performance and competitiveness (Corvello et al. 2021; Secundo et al. 2020). As for the external environment, digital technologies modify the processes through which SME entrepreneurs evaluate the environment in which they operate, identifying new opportunities and defining new strategies (Dellermann et al. 2020; Scarmozzino et al. 2017).

With the pandemic emergency, this phenomenon has been significantly amplified, as smart working has become the primary way of interaction (Contreras et al. 2020) with a consequent increase in the use of digital technologies and an improvement in people's digital knowledge (Manco-Chavez et al. 2020). More in general, the use of digital technologies for dealing with the consequences of extreme events, such as COVID-19, has been recently investigated (Papadopoulos et al. 2020). Amankwah-Amoah et al. (2021) argued that COVID-19 has evolved to be a kind of “catalyst” for the adoption and increasing use of digital technologies in business.

With reference to the reaction of organizations to crises, the role of digital technologies has been studied at strategic and operational level.

At the strategic level, it has been observed that digital technologies can make it easier for managers to analyze the context made turbulent by the crisis (Acciarini et al. 2021). With specific reference to SMEs, Audretsch and Belitski (2021) have found that digital technologies positively affect performance in times of crisis because they support the acquisition and management of complex knowledge.

At the operational level, digital technologies can improve collaboration and flexibility, both internally and at the supply chain level, since they facilitate information sharing, standardization and recombination of procedures (Samvedi et al. 2013) as well as increase availability of data and efficiency of communication (Buyukozkan and Gooçer 2018, p. 165). Brandon-Jones et al. (2014) suggest that supply chain connectivity and information sharing resources lead to a supply chain visibility capability which enhances resilience and robustness. Dubley and colleagues discussed on data analytics capability as a means to improve information-processing capacity and supply chain resilience (Dubley et al. 2021).

Therefore, digital technologies influence the resilience both at firm and at supply chain level because they contribute to increase flexibility, to create redundancy, to form collaborative relationships and to improve knowledge acquisition and transfer.

However, Nikokar and colleagues have noted that the search for robustness also involves risks: robust firms sometimes end up succumbing to catastrophic events. They highlight that, while the ideas of resilience and robustness impliy the desire to return to the status quo prior to the event that triggered the disorder, antifragility is the ability to gain from disasters. Digital technologies, if properly designed, make it possible to create spaces of redundancy and creative recombination of existing resources, necessary to develop antifragility (Nikokar et al. 2021).

In summary, digital technologies have a pervasive role in SMEs, even more so after the crisis for COVID-19, to the point of influencing every aspect of their functioning. Since digital technologies have a positive impact on resilience, it is therefore to be expected that they will also play a fundamental role in the development of antifragility. As far as the authors are aware, however, there are still no specific study on the relationship between digital technologies and antifragility in SMEs.

## Methodology

### Research design and sample

The methodology used for the research is the multiple case study approach. This approach is generally used to answer questions such as ‘who’ and why in a real-world context (Yin 2014) as well as in theory building (Eisenhardt and Graebner 2007). Furthermore, this methodology allows obtaining the effect of mitigating the validator’s judgments and on the other hand increasing the external validity (Eisenhardt and Graebner 2007; Yin 2014). Findings of a case study are not expected to be widely generalizable but to contribute more substantially to the formulation of new hypotheses and to enable subsequent investigations according to other research designs (Sellitto 2018).

Six cases of SMEs located in Southern Italy were selected in order to obtain a higher amount of variation (Flyvbjerg 2006). To identify SMEs the parameters set by European Union were used. With reference to the core business activity, both digital SMEs and traditional companies were analyzed, in order to understand how digital technologies influenced the two different types of organizations in the response to the pandemics. The participating organizations were identified through media and internet information and personal networking.

A summary of main features of the firms in the sample is reported in Table 1. Six small and medium firms were selected for the study.

**Table 1:** Main features of cases study

Startup	Sector	Product description	Interviews number and duration	Other sources of data
Revelis	ICT	AI and big data	1 interview, 60 min	Website
Lanificio Leo	Manufacturing	Luxury textile product	1 interview, 60 min	Website; Workshop
FMB Tubes	Metallurgical	Metal carpentry, steel and iron structures, prefabricated boxes	1 interview, 60 min	Website
Macingo	Transport	Buying and selling freight transport	1 interview, 60 min	Website
Altrama	ICT	Website, portals, ecommerce, apps, web editorial contents	1 interview, 60 min	Website; Workshop
Internet & Idee	ICT	Information systems	2 interview, 60 and 30 min	Website; Internal documentation; Workshops

### Data collection

Data were collected in May 2021. In total six interviews were conducted. The interviews lasted about 60 min and were conducted in conference calls using a semi-structured questionnaire. The questionnaire included two sections: the first section focused on structural characteristics of the SME (e.g. number of employees, revenues, year of foundation), while the second section about different topics: challenges and results during pandemic (antifragility experience), digital technologies (interaction with environment and role in internal transformation) and contingent variables to firm’s antifragility. Interviewees were people with extensive knowledge of the SME (in most of the cases the entrepreneur), with an apical role in the management of the organization. Interviews were recorded, transcribed and a report was written for each case study; moreover, the feedback from the interviewees on the questions was used to modify the protocol in later interviews. In case of unclear or missing information, the interviewees are contacted for clarification.

### Data analysis

The analysis of the case studies was carried out in two phases: first, the analysis of the single case study was carried out, second, a cross case analysis was carried out to integrate the results of all six cases and additional data.

More specifically, in the first phase a content analysis of the individual cases was carried out with the application of an approach deriving from grounded theory (Strauss and Corbin 2008). The interviewees were carried out separately by at least two authors to make a comparison and discuss any case of divergent interpretations. A codification of the interviews was realized to identify the relevant concepts (Pratt 2009) and the relationships between them. First-order concepts were grouped together in second-order themes that describe data at higher lever (Clark et al. 2010). Finally, the second level concepts have been grouped into three aggregate dimensions (Silva et al. 2021). In the second phase, the results that emerged from the within-case analysis were compared with each other through a cross-case analysis to further modify the concepts identified in each of the three levels in the first phase until reaching their final version with an acceptable degree of internal coherence, between cases and data adaptation. The results obtained with the within case and with the cross-case analysis were then further analyzed in order to identify similarities and differences between the case studies.

## Results

In the next paragraphs each case study is discussed individually. For each firm in the sample, a description of its core business, the experience of antifragility and relative results, the digital tools used (in terms of environmental interaction and internal transformation) and principal contingency variables of antifragility are discussed. In the following section the six cases are compared again with respect to the same elements of analysis listed above.

### Within case analysis

#### Revelis

##### General description of firm

*Revelis* is an innovative start up operating in Artificial Intelligence and Big Data Management field. The firm operates in many different markets: manufacturing, banking and finance, energy and utilities, telecommunication/contact center, logistics and transport, public administration, health and well-being of people, retail. The company was founded in 2019, it has grown rapidly in just 2 years from 3 to 16 employees and achieving in 2020 a turnover of about 900.000 €.

##### Antifragility experience and digital tools

The company’s antifragility capacity during the COVIDCoViD-19 pandemic is represented by the development in that period of a specific product, called ‘*ai-guard’*, that verifies compliance with anti-COVID regulations with neural networks. The product was launched on the market in October 2020 and is slowly being introduced in various contexts. During the interview, the CEO focused his attention also on the positive effects on the firm of the massive use of smart working in the last year. Remote working has increased the firm competitiveness through the reduction of costs associated with travel to customers. Moreover, the widespread use of call conferences and greater emphasis on the social side, have allowed the firm to reach a greater number of target customers than the pre-pandemic period. The use of digital technologies has supported the firm both with external relationships and in internal transformation, by affecting all four variables investigated in this research (visibility, communication, intelligence and process support), both in qualitative and quantitative terms. Therefore, referring to the three contingency variables for antifragility, the entrepreneur stated that ‘finance’ was certainly the fundamental one to overcome the pandemic period.

#### Lanificio leo

##### General description of firm

Lanificio Leo, founded in 1873 is a significant cases of a company-museum, in fact, it has a collection of historical machinery ranging from 1980 to 1965, which are still the beating heart of the company, continuously integrated with the latest generation machinery. Therefore, the firm represents a mix of a traditional know-how and a strong propensity of innovation.

##### Antifragility experience and digital tools

The COVID-19 crisis has pushed the firm to an important acceleration in its digital presence particularly on luxury international marketplaces. Therefore, the firm focused on reengineering and restyling of the firm website. All these digital activities allowed the start of new important partnership. The lockdown led to closure of the main store located at the Lamezia Terme international airport, thus causing a worsening turnover in 2020. However, the digital alternatives put in place during the pandemic, should lead to a strong increase in revenues in the next year. The web has not only a way to open a new potential sales channel, but it has been also crucial to acquire new important customers despite the crisis. An example is ‘*La Rinascente*’ with which during the pandemic the firm managed to conclude an agreement for the sale of its products in their stores. The use of digital technologies was fundamental in this case in terms of environment interaction, in particular the right use of these tools has allowed the firm to quickly involve people that the entrepreneur had never physically met, creating in this way new important partnerships. With reference to the four variables analyzed in the interview, the entrepreneur stated that digital technologies do not have effects on process support. Referring to the contingency variables, the CEO underlined the importance of the previous investment in industry 4.0, his creativity and the proactivity of his team for overcoming the crisis period from COVID-19 emergency.

#### FMB Tubes srl

##### General description of firm

*FMB Tubes srl* for over 20 years is a point of reference in Calabria and in Italy in the field of metal carpentry, construction of steel and iron structures and of prefabricated boxes for companies and individuals. The company’s operational activity takes place on two production plants with the support of a team of specialized technicians. The firm has an annual production capacity of over 20,000 tons of steel and a growing turnover. In particular, in 2020 turnover is increased of 15% compared to the 2019, reaching 6 million euros, with growth prospects also for 2021.

##### Antifragility experience and digital tools

During the pandemic, the firm managed its competitive advantage, which is a result of previous investments in terms of digitalization and modernization of machinery, obtaining a very high number of orders for modular buildings throughout Italy. This allowed the firm to double the turnover of the modular prefabricated line. The digital technologies have affected both the environmental interaction and internal transformation. In particular, instant messaging apps have become the principal communication tool in the work team. In terms of visibility and external communication, the very important tools are social network and browser (above all SEO indexing). Other important elements are analytics tools for intelligence and process support and finally BI databases are strategic in terms of intelligence because they allow the firm to find information on potential customers. With reference to contingency variables, the interviewee stated that of the three variables indicated for the antifragility, only finance was fundamental because ‘*having the accounts in order was certainly a plus compared to the competitor*’.

#### Macingo

##### General description of firm

*Macingo* is an online platform, which connects customers who need to transport bulky goods with suitable carriers for this activity. The company employs a team of 14 people including co-founders, software architect, developer, SEO manager, some social media marketing and logistics specialists. In terms of business, over the last year, the platform has received an average of 25.000 transport requests per month, reaching a turnover of over 1.500.000 €, with an increase of 50% compared to 2019.

##### Antifragility experience and digital tools

The pandemic was a business accelerator; there was a sharp increase in transport requests, especially from private individuals, who asked for delivery to their home (while pre-COVID delivery took place at a collection point). The efficiency of the services offered by the firm has also made it possible to start two important collaborations: the first with Decathlon (for the delivery of treadmills to customers) and the second a partnership with a French refrigerated transport company. The COVIDCoViD-19 pandemic was for Macingo an opportunity; in fact, it had a 15% increase in margins and a high increase of traffic on the website. In reference to the use of digital technologies, the interviewer stated that they have an impact on communication, visibility, and process support. As regards the contingency variables, for the firms it was very important two elements: ‘*finance’* and *‘internal skills*’, having a positive cash flow and specific skills made it possible to overcome lockdown and the pandemic without too much difficulty.

#### Altrama

##### General description of firm

Altrama is a software house that offers customers services to create and manage web-oriented software solutions, promote online activities of public and private entities and improve their work processes with the support of AI (artificial intelligence). Altrama designs and implements websites and portals, eCommerce and apps, creates web editorial contents optimized for search engines and manages web portals such as ViaggiArt and ANSA ViaggiArt.

##### Antifragility experience and digital tools

In this case, the antifragility is represented by the stability of the turnover, in fact the firm maintained in 2020 the same turnover of 2019 (about 400.000 €), despite the firm’s core business being linked to the tourism sector which has completely stopped with the pandemic. Another important aspect was the firm’s branding activities that have brought contacts and relationships with new customers. The use of digital technologies has impacts on intelligence, communication and visibility both internal and external level. Referring to contingency variables, the interviewer stated that the most important was finance and external networks that were the principal elements that have allowed the firm to move forward even during the pandemic.

#### Internet & Idee

##### General description of firm

Internet & Idee is a medium sized ICT consultancy company, developing information systems for customers in the apparel and creative industries. It works on project-based fashion. The company grew rapidly in the past few years from less than 20 employees to over 80, achieving a turnover of 5 million euro in 2019.

##### Antifragility experience and digital tools

During the interviews, the entrepreneur repeatedly stated that the period of the pandemic has been an opportunity for exploring new markets, looking for new technologies, and evaluating the opportunities they create for the firm. Indeed, the company had developed a competence in working from a distance and, as the lockdown made this the main way of working, this experience represented a competitive advantage. Working with customers became easier and they found in *Internet & Idee* an efficient partner. During the last 3 years, the use of digital tools has increased. In particular, desk video call is the tool which showed the strongest increase in usage, even before the COVID-19 pandemic. Industry databases are used strategically by the entrepreneur when important decisions are made. During the last months new, medium-term contracts have been closed and new personnel has been hired.

Table 2 summarized the main results of data collection for each case study.

**Table 2:** Main results of data collection

Main results	Revelis	Lanificio Leo	FMB Tubes	Macingo	Altrama	Internet & Idee
Core business before the crisis	Product based on Artificial Intelligence	Luxury Texile Products	Metal carpentry Prefabricated box	Bulky goods transport	Website, portals, ecommerce, apps, web editorial contents	Information systems
COVID-19 opportunities	Product for compliance with social distances measures	New digital sales channel	Strong demand for prefabricated boxes	New partnerships and a sharp increase in the demand for transport	Acquisition of new customers and consequently creation of new relationships	Exploration of new markets
Aspects of internal context influenced by digital tools	Intelligence Communication Visibility Process support	Intelligence Communication Visibility	Intelligence Communication Visibility Process support	Communication Visibility Process support	Intelligence Communication Visibility	Communication Process support
Aspects of external environment influenced by digital tools	Intelligence Communication Visibility Process Support	Intelligence Communication Visibility	Intelligence Communication Visibility Process Support	Communication Visibility Process Support	Intelligence Communication Visibility	Intelligence Communication Visibility
Contextual factors	Finance Internal skills	Finance Internal skills External network	Finance	Finance Internal skills	Finance External network	Finance External network

### Cross-case analysis

#### Nature of antifragility in the investigated SMEs

The companies we investigated did not stop investing and pursue new opportunities during the pandemics. Instead, they rapidly modified characteristics of their businesses and, as far as can be said so far, improved their strategic position. They acquired new customers, entered new markets, and accessed new distribution channels. Macingo and Altrama, for example, strengthened their position in market segments they were operating in but that were not part of their core business. Revelis developed new products. Lanificio Leo sped up the process of entering a new distribution channel. All the interviewees underline that the opportunity seeking attitude is a necessary consequence of the entrepreneurial nature of their organizations. Either because they are young firms, or they operate in a rapidly evolving market (e.g. software development) or they have undergone recent radical transformations, they have a positive attitude towards change. For example the entrepreneur in Lanificio Leo stated:

“The company is old, but it underwent a radical transformation with the last generation of owners. It can be considered a startup company still looking for a stable business model”.

#### The role of contextual factors

The development of antifragility in SMEs appears to be influenced by several factors. Among these, the following are often cited by the interviewees: (a) the availability of financial resources; (b) a specific combination of internal skills; (c) access to a network of external skills and resources.

Of the six interviewees, five spontaneously mentioned financial resources as one of the factors that helped them to improve their situation during the crisis. The six confirmed the importance of this factor after direct question by the interviewer. From the comments of the interviewees, it emerges that the availability of financial resources had a double effect: (1) it provided both the material means for starting new projects and (2) it gave entrepreneurs the psychological tranquility to start a new initiative.

For example, in the case of Macingo during the interview emerged that:

“The positive cash flow of the company has certainly made it possible to overcome the first lockdown period without too many problems and giving to the firm the peace of mind to focus on the deployment of specifically internal skills when new business opportunities arise”.

External resources were often important for integrating the internal capabilities of firms in adjusting the newly developed processes. Several times during the interviews it was observed that it would have taken much longer to develop new products and processes working in isolation. External resources like universities, partner components, industrial customers provided complementary resources and competences. In some cases. these external actors seem to have been permanently included in the value chain of the focal company. For example, the entrepreneur in Revelis explained that:

“Relations with external network were important during the pandemic, but they are a fundamental focus of the company life regardless of the crisis. We have constant relationships, for example, with the University of Calabria, the University of Palermo and the CNR. Research projects are for us not only an important financing asset, but also an opportunity to maintain relationship with these partners”.

In other cases, they contributed to manage the transition towards a new business model while the focal company was acquiring or developing the needed resources.

The role of financial resources and external networks is especially relevant when digital technologies are involved; this is particularly true for those companies which adopted these technologies only recently: the transition towards new digitally enabled processes or business models was better managed when financial resources were available and when external networks were strong. As the interviewed entrepreneur of Lanificio Leo stated:

“Customers outside the local market have always been essential for the life of the firm. The open attitude to innovation, to explore new fundamental solutions of R&D, is a resource to draw on. In some cases, it's not as powerful as a financial asset, but the speed with which you can change the vision is especially critical in small businesses”.

#### Digital technologies and antifragility

According to respondents, digital technologies played an important role in how the pandemic was dealt with. They were used as a tool to (a) interact with the environment; (b) understand the changes in progress in a timely manner; (c) gain visibility for one's own initiatives and only to a lesser extent (d) to manage internal work, coordinating with employees or to automate processes.

Interviewees perceive themselves as proficient with digital technologies. This is justified in some cases by the fact that they operate in a digital market (e.g., in the case of Altrama, Internet & Idee or Revelis). In other cases, they had recently started new projects involving digital technologies. Interviewees often suggest that the use of digital technologies in their organization is fluid and not made rigid by consolidated routines. As one of the interviewees stated:

“When digital technologies are used in an easy way within the company, for example to solve organizational problems related to physical distance, they become a real complementary tool for business activity”.

A diffuse observation among the interviewed companies, is that all were in a situation in which, because of different reasons, digital processes were fluid, that is, not structured into rigid routines. In some cases, it depends on recent change of business model, like for Macingo, in other cases on the youngness of the firm, like for example for Altrama. In any case the fluidity of processes was associated by interviewees with the ability to face the crisis.

It is interesting to note the difference between the interviewed companies operating in a market that offers digital products and services, compared to organizations with mainly physical products or services. In the first case, it emerges from the interviews that, beyond an initial phase of concern, there was no perception of substantial change in the business activity. Entrepreneurs continued to seek opportunities by offering customers solutions suited to the new environment, but without a change in the nature of the business. However, in the second case, a change of course was perceived. In fact, these companies historically have a corporate focus based on quality and material elements (such as the products they manufacture and sell), but with the advent of the pandemic crisis they focused on intangible elements such as digital services. For example, Lanificio Leo enhanced its presence on international digital channels. FMB tubes, has also invested in the digital services for its clients. This is evidently due to the nature of the current crisis, which has made it difficult to move physical goods and has increased the demand for digital services. But the phenomenon was also perceived as the acceleration of a pre-existing and widespread transition towards the immaterial dimension.

Another interesting element is that the interviewees report having perceived a different attitude towards digital technologies in the external environment. It emerges from the statements of many, that they believe they have made efforts in the past to embrace digital transformation and how the pandemic has rewarded their efforts as it has accelerated the adoption of these tools by customers and partners.

Table 3 reports, in a schematic way, how the results of the paper allow to reduce the gap present in the literature.

**Table 3:** Reduction of literature gaps through research results

Literature gap investigated in this paper	Research results
Research on antifragility in SMEs is still limited	Refinement of our understanding of antifragility through the analysis of the reaction to the crisis in the six cases^a^
Antecedents of antifragility in SMEs are unclear (RQ1)	Three main antecedents of antifragility Slack financial resources Strategic agility Networking with research institutions
The role of digital technologies in building antifragility is unclear (RQ2)	Digital technologies were used as a tool to (a) Interact with the environment (b) Understand the changes in progress in a timely manner (c) Gain visibility for the SMEs’ initiatives (d) Coordinating with employees or automating processes

^a^See also Table 2

## Discussion

The aim of this paper is twofold, mirroring the two research questions outlined in the introduction. On the one hand it has the objective to investigate the antecedents of antifragility in SMEs. On the other hand, given the role digital technologies play in every aspect of the functioning of contemporary businesses and the consequent expected impact on antifragility, the paper specifically aims at deepening our understanding of the influence of digital technologies on the development of antifragility in SMEs.

**Fig. 1:**
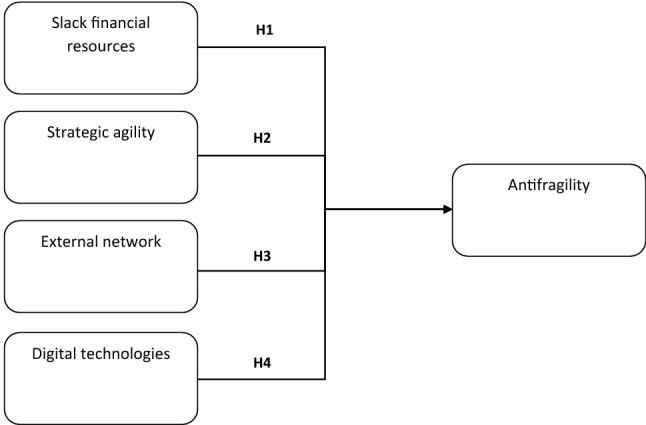
Proposed research framework

The paper has an exploratory nature (Eisenhardt and Graebner 2007). In this paragraph results will be discussed and hypotheses to be analyzed in future studies will be proposed (Fig. 1).

With reference to the first research question, in most of the interviews, the entrepreneurs associated antifragility with the availability of resources, both tangible and intangible, which create a certain margin of maneuver for the company and allow to manage the transition through the crisis. Financial resources, in particular, were cited by all respondents. By comparing our data with the results of the previous literature (Klein and Todesco 2021; Lafuente et al. 2017), we can conclude that, in addition to being necessary for new investments, financial resources during crises also create the psychological conditions to look for new business opportunities. We can formulate the following hypothesis:

H1. Slack financial resources have a positive impact on antifragility.

The attitude towards opportunity seeking is a necessary component of antifragility. All respondents pointed out that for their organizations the crisis was perceived not very differently from the common difficulties they face and overcome. This attitude is part of the nature of entrepreneurial firms: they consider the environment as a source of threats and opportunities and their responsibility is to find a feasible path for survival (Branicki et al. 2018). When discussing antifragility, such an attitude is often associated by the interviewees with the flexibility of the internal organization. In particular, internal processes are described as fluid (Mamouni Limnios et al. 2014). This combination of entrepreneurialism and flexibility is consistent with the definition of (strategic) agility (Ahamd et al. 2020; Ramaezani and Camirinha-Matos 2020), in particular with reference with SMEs (Troise et al. 2022). The interviewees, then, describe their organizations as strategically and operationally agile. Based on these observations we can formulate the following hypothesis:

H2. Strategic agility has a positive impact on antifragility in SMEs.

A third factor which proved to be relevant for building antifragility in the investigated SMEs, is external networks. Networks are important to access resources not available internally (Jespersen et al. 2018). A wide network of relations allows access to a wide range of resources (Lazzarotti et al. 2017). Results from our interviews confirm the importance of having a solid network of external partners. This enables SMEs to build more competitive businesses in the long run, but it is also important to rapidly compensate for the lack of internal resources during a crisis, especially in the case of resources which are needed rapidly or only for a short period. Therefore, we can formulate the following hypothesis:

H3. The breadth and depth of external relationships have a positive impact on antifragility in SMEs.

The second research question focuses on a specific type of antecedent of antifragility in SMEs: digital technologies. The literature suggests that the adoption of digital technologies has a positive impact on SMEs adaptation in complex contexts (Skare and Soriano 2021). Similarly, our findings support the idea that the adoption of digital technologies facilitates the development of antifragility. All the interviews confirm that digital technologies played a central role in the adaptation processes of their organizations. We can formulate, then, the following hypothesis:

H4. The adoption of digital technologies is positively related to antifragility in SMEs.

However, the relationship between these two variables as described by the interviewed entrepreneurs, is complex. It is not limited to digital technologies enabling antifragility. Based on the observation from the interviews, it emerges that for entrepreneurs digitalization represents first of all a dimension and a context for their work. This means that, at least in part, the daily activity of entrepreneurs and employees takes place in the digital environment as once it took place in the real world. Firms perceive a digital dimension in their activities and those firms able to navigate this context prove to be more antifragile. In other words, as pointed out by previous studies (Depaoli et al. 2020), the link between digital technologies and antifragility seems to rely on the intelligence of the digital world. On the other hand, digital communication, defined as the capability of communicating through digital channels, appears to be important for the value created to be visible for the customer (Park and Mithas 2020). By supporting context intelligence as well as visibility of the firm in the complex context of large crises, digital technologies positively affect antifragility (see Table 3).

## Conclusion

The COVID-19 pandemic has faced a huge challenge for small and medium-sized businesses (Zimmerling and Chen 2021). Crises, even if of a smaller scale, are not uncommon in the life of an organization. While some businesses succumb to difficulties, others thrive (Ramezani and Camarinha-Matos 2020). This study analyzes the conditions in which SMEs show antifragility, that is, they are able to thrive in times of crisis.

The property of antifragility is studied in this article in relation to the context of digital transformation of the economy that companies are experiencing (Garzoni et al. 2020).

The results of the article suggest that digital technologies favor the development of antifragility. The relation between digital technologies and antifragility in SMEs appears to be not linear. The view of digital technologies as enabling antifragility is limited and probably misleading. Digitization represents a context in which small and medium-sized enterprises must learn to navigate. That is, they must become digitally competent. In a fluid context, such as the digital one, this facilitates agility even in the face of crises (Manyati and Mutsau 2021).

Our study highlights how the link between competence in digital technologies and antifragility is strengthened by the presence of slack resources (Tognazzo et al. 2016) that allow small and medium-sized enterprises to have room for maneuver in adapting to change.

This article has limitations. The number of companies analyzed is compatible with the goals of the research and coherent with the suggestions by Eisenhardt (1989), who suggested the ideal number of case studies being between four and ten. A larger sample can increase the generalizability of the results. Furthermore, only firms showing antifragility characteristics were studied. It would be useful in the future to compare antifragile firms with firms that have not demonstrated this property.

The companies analyzed are all service companies. The conditions under which antifragility occurs may be different in industry.

All the companies analyzed are in the same territory. An analysis comparing companies from different geographic areas could increase the validity of the results.

Our analysis only hypothesizes direct relations among the variables. Mediation and moderation effects are also possible. For example, agility could be a mediator between the other variable, digital technologies in particular, and antifragility. On the other hand, financial resources or external networks could moderate the relation between digital technologies and antifragility. Such hypotheses should be tested in future quantitative studies.

Finally, the companies were analyzed close to the crisis. If this allows us to have timely information on the strategies adopted for antifragility, it does not allow us to measure the medium-long term results of the proposed initiatives and, consequently, to affirm with certainty that the competitive position of companies has improved.

From a research point of view, this article provides a series of propositions linking digital technologies, business skills in this area and antifragility. Future research can test these hypotheses in a large sample of small and medium-sized enterprises, helping to improve our understanding of the phenomenon.

From a managerial point of view, this research provides a new and more articulated vision of the link between digital technologies and antifragility. Digital technologies are not sufficient in themselves. They are not instrumental in generating antifragility. They represent a context of action and decision for entrepreneurs who must become competent in navigating the digital environment, in which a large part of business activity takes place today (Priyono et al. 2020). This expertise enables companies to quickly identify new opportunities and modify their processes to seize them.
